# The effect of levodopa on speech graphs in non-demented patients with multiple system atrophy and Parkinson’s disease

**DOI:** 10.3389/fneur.2026.1746101

**Published:** 2026-02-09

**Authors:** Jingyue Liu, Yan Zhao, Jinghong Ma, Guanyu Zhang

**Affiliations:** 1 National Clinical Research Center for Geriatric Disorders, Xuanwu Hospital of Capital Medical University, Beijing, China; 2 Department of Rehabilitation Medicine, Jinan Third People’s Hospital, Jinan, China; 3 Department of Neurology, Xuanwu Hospital of Capital Medical University, Beijing, China; 4 China Institute of Sport Science, Beijing, China

**Keywords:** cognition, levodopa equivalent daily dose, multiple system atrophy, semantic fluency, speech graphs

## Abstract

**Background:**

An objective topological analysis for any arbitrary network was provided by graph theory, which was implemented to reveal topological properties of brain networks in a wide range of neurodegenerative diseases.

**Objectives:**

To investigate the difference of the topology of speech graphs among parkinsonian-type multiple system atrophy patients (MSA-P), idiopathic Parkinson’s disease (PD) patients, and healthy controls (HC). To explore the effect of levodopa on the topology of speech graphs in MSA-P and PD patients.

**Methods:**

We applied a graph analysis of topology of speech graphs in MSA-P patients (*N* = 39), idiopathic PD patients (*N* = 51), and HC (*N* = 51). All participants were assessed by a well-established semantic fluency test (animal). Participants’ verbal reactions were listed and represented as speech graphs with directionality.

**Results:**

MSA-P patients mentioned more about their memory and remaining time than PD patients (*p* < 0.001) and HC (*p* < 0.001). Moreover, MSA-P patients’ speech graphs exhibited fewer nodes (PD: *p* = 0.004; HC: *p* < 0.001), higher density (PD: *p* = 0.007; HC: *p* < 0.001), smaller diameter (PD: *p* = 0.004; HC: *p* < 0.001), and smaller average shortest path (PD: *p* = 0.002; HC: *p* < 0.001) than both PD patients and HC. Compared with PD patients and healthy adults, the MSA-P patients generated denser and smaller speech graphs. Importantly, in MSA-P, the positive correlations between levodopa equivalent daily dose and diameter (*r* = 0.39, *p* = 0.017) and average shortest path (*r* = 0.38, *p* = 0.017) were obtained, suggesting that higher levodopa doses were associated with producing sparser and larger speech graphs in MSA-P patients. However, there was no significant correlation between levodopa equivalent daily dose and graph indices in PD patients.

**Conclusion:**

MSA-P patients’ speech graphs were denser and smaller than both PD patients and healthy adults. The graph analysis of semantic fluency can differentiate MSA-P from PD to some extent. Importantly, in MSA-P patients, the impaired speech topology can be partially restored by levodopa.

## 1 Introduction

Multiple system atrophy (MSA) is defined by striatonigral and olivopontocerebellar degenerations with various combinations of autonomic, extrapyramidal, and cerebellar symptoms (1). MSA has many clinical phenotypes, such as parkinsonian-type MSA (MSA-P) and cerebellar-type MSA (MSA-C). Usually, it has difficulties in differentiating between early-stage MSA-P and idiopathic Parkinson’s disease (PD), because they share similar clinical features (2). Therefore, a effective tool to differentiate MSA-P from idiopathic PD is clinically meaningful.

The well-established semantic fluency test has been frequently used measure in research and clinical practice for assessing the ability to produce as many different words of a specific category (e.g., animal) as possible during one minute (3). Many parameters have been proposed for measuring the performance of this test, for example, the count of correct words of MSA or PD patients was lower than healthy adults (4, 5). Clustering and switching analyses are also common, which defined two key parameters based on the similarity or closeness of word meaning (6). The average count of correct words with high semantic relatedness (e.g., invertebrate animals) is defined as the mean cluster size, and the count of cluster switches. Compared to healthy adults, the PD, Alzheimer’s disease (AD), or progressive supranuclear palsy (PSP) patients exhibited fewer clusters and switches in verbal fluency tests (7, 8). However, this approach obtains limited information and depends primarily upon subjective estimation of cluster division. An automated classification approach conflicted with classic experimenter-based method in evaluating clusters (9).

An objective approach originates from graph theory, which calculates network properties to provide an assessment of network structure and changes. The graph theoretical analyses have been implemented to depict the topological dynamics of brain networks in a variety of neurodegenerative disorders (10, 11). Similarly, this approach have been used to analyze semantic fluency data. Our previous studies found that AD patients’ speech graphs exhibited fewer nodes, higher density, smaller diameter, and smaller average shortest path than PD patients (12), which was same as the comparison between PSP patients and PD patients (13), suggesting that the cognitive decline associated with denser and smaller speech graphs.

This study used graph theoretical analysis to investigate the speech topology of MSA-P patients. All participants were assessed by a well-established semantic fluency test (animal). Their verbal reactions were listed and represented as speech graphs with directionality. A node represents a correct word and an arc represents the temporal connection between sequential words. First, group differences in meta references (metalinguistic reference and metacognitive reference) and graph indices (nodes, density, diameter, and average shortest path) were examined. Second, the effects of levodopa on meta references or graph indices were explored in MSA-P and PD, respectively.

## 2 Materials and methods

### 2.1 Patients and clinical assessments

We included 39 patients with MSA-P (Movement Disorder Society Clinical Diagnostic Criteria for Multiple System Atrophy (14)) at the Xuanwu Hospital of Capital Medical University between 2024–2025.

All patients were evaluated on their regular anti-parkinsonian drugs, including levodopa (*N* = 31), amantadine (*N* = 17), pramipexole (*N* = 11), stalevo (*N* = 9), selegiline (*N* = 4), rasagiline (*N* = 2), and benzhexol (*N* = 1). Anti-parkinsonian drugs were calculated according to the formula of Tomlinson et al. (15) that yielded a total levodopa equivalent daily dose. The Movement Disorder Society sponsored revision of the Unified Parkinson’s disease Rating Scale (MDS-UPDRS) was expanded upon the original UPDRS by including more non-motor items, making it become a robust tool to evaluate the severity of motor (Part III subscale) and non-motor symptoms (Part I subscale). Demographic and clinical assessments and neuropsychological features were demonstrated in Table 1. For comparison between the MSA and PD groups, there was no difference in the MoCA (*p* = 0.294), even though the difference among three groups was significant.

**Table 1:** Demographic and clinical assessments, and neuropsychological features of patients and healthy controls (means, standard deviations, ranges, and group differences).

Assessments/features	MSA-P (*N* = 39)	PD (*N* = 51)	HC (*N* = 51)	Group differences (*p* values)
Male: female	18:21	26:25	24:27	0.883
Age (years)	61.2 (7.8) 45–79	59.2 (10.1) 41–80	59.0 (7.6) 40–74	0.439
Education (years)	11.4 (3.3) 5–19	12.3 (3.2) 6–18	11.9 (2.2) 7–16	0.373
Motor symptoms				
MDS-UPDRS III: motor examination	36.9 (14.1) 4–66	32.6 (11.4) 12–55	—	0.117
Hoehn and Yahr scale	2.3 (0.7) 1–3	2.1 (0.6) 1–3	—	0.103
Disease duration (years)	0.9 (1.2) 0–5	1.3 (1.5) 0–5	—	0.124
Duration of motor symptoms (years)	2.4 (1.2) 1–6	2.6 (2.0) 0–9	—	0.535
Levodopa equivalent daily dose (mg/day)	388.2 (224.5) 25–875	313.2 (229.4) 38–1,000	—	0.124
Non-motor functions				
MDS-UPDRS I: non-motor experiences of daily living	11.4 (5.6) 2–24	9.9 (4.6) 3–21	—	0.166
Beck depression inventory-II	4.2 (2.8) 0–7	3.3 (2.0) 0–7	3.3 (1.7) 0–7	0.105
Epworth sleep scale	4.1 (2.7) 0–10	3.9 (3.7) 0–14	3.4 (2.5) 0–10	0.536
Montreal cognitive assessment	24.9 (2.4) 21–30	25.4 (2.6) 21–30	27.9 (1.4) 26–30	<0.001*

MSA-P, parkinsonian-type multiple system atrophy; PD, Parkinson’s disease; HC, healthy controls; MDS-UPDRS, the Movement Disorder Society-sponsored revision of the Unified Parkinson’s disease rating scale; Group differences, *p* values of one-way ANOVAs or Kruskal-Wallis one-way ANOVAs as appropriate; asterisks (*), a significant group difference.

### 2.2 Two control groups

This study recruited two control groups: 51 age, sex, and education-matched idiopathic PD patients [Movement Disorder Society Clinical Diagnostic Criteria for Parkinson’s disease (16)] from Xuanwu Hospital of Capital Medical University and 51 age, sex, and education-matched healthy controls (HC) from nearby communities.

For the PD group, they were evaluated on their regular anti-parkinsonian drugs, including levodopa (*N* = 37), pramipexole (*N* = 17), selegiline (*N* = 11), piribedil (*N* = 10), amantadine (*N* = 5), and entacapone (*N* = 3). As MSA-P patients, they completed the same neuropsychological and clinical measures.

For the HC group, they completed the same measures for mood, sleep, and cognition as patients.

### 2.3 The inclusion and exclusion criteria

For all groups, inclusion criteria were (1) age 40 to 80 years; (2) education ≥5 years; (3) Mandarin Chinese speaking (native or fluent Mandarin Chinese speakers). Exclusion criteria were (1) intake of anti-depressants or potential current depression (Beck Depression Inventory-II, BDI-II > 7); (2) a history of brain injury or neurological or psychiatric disorders or inherited diseases; (3) drug or alcohol abuse.

For MSA-P and PD groups, inclusion criteria added Hoehn and Yahr Stages 1 to 3 and exclusion criteria added intake of anti-dementia drugs or potential current dementia (Montreal Cognitive Assessment, MoCA<21/30). Particularly, there was no MSA-P patient who did not receive levodopa (levodopa equivalent daily dose = 0 mg/day).

For HC group, exclusion criteria added intake of anti-dementia drugs or potential current mild cognitive impairment or dementia (MoCA<26/30).

### 2.4 Meta references and graph indices

All participants were assessed by a well-established semantic fluency test (animal). Their verbal reactions were listed.

The two meta references were: (1) metalinguistic reference: the count of times participants mentioned about words they generated (e.g., “Is it right to say shrimp?”); (2) metacognitive reference: the count of times participants mentioned about their ability to complete this task or asked remaining time (e.g., “I can’t do this.”).

**Figure 1:**
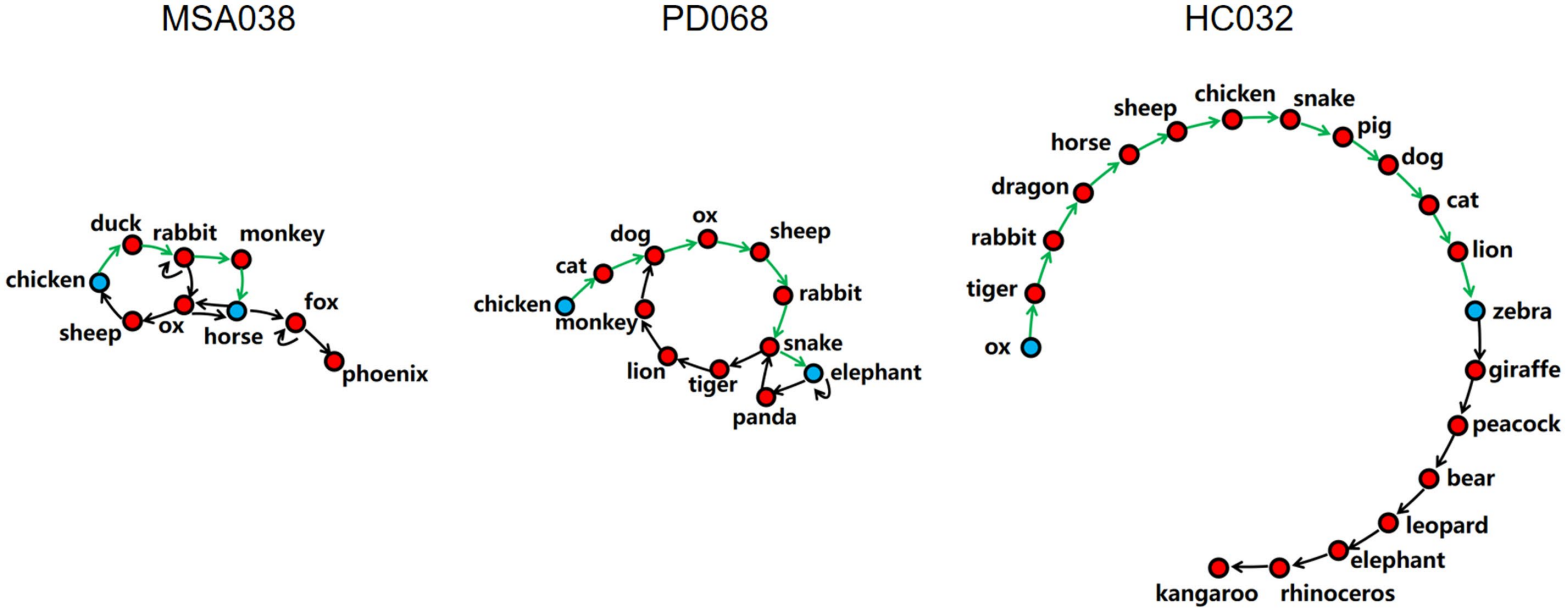
Diagram of speech graphs with directionality of three typical participants: MSA038, a parkinsonian-type multiple system atrophy patient; PD068, a idiopathic Parkinson’s disease patient; HC032, a healthy control subject. Examples of the shortest path (green) between two nodes (blue) in the three speech graphs with directionality. The nodes represent correct words. The arcs represent temporal connection between nodes. The density is the ratio of the count of arcs to the maximum possible count of arcs. The diameter is the length of the longest shortest path. The average shortest path is the mean length of all the shortest paths. It can be clearly seen from the figure that the speech graphs of three participants from three different groups became sparser and larger in sequence.

Participants’ verbal reactions were represented as speech graphs with directionality by Speech graph software (12, 13), in which a node represents a correct word and an arc represents the temporal connection between sequential words (Figure 1). We calculated four graph indices, including the nodes, density, diameter, and average shortest path. The correct words are the nodes. The ratio of the count of arcs to the maximum possible count of arcs is the density. The length of the longest shortest path between two nodes is the diameter. The mean length of all the shortest paths between two nodes is the average shortest path.

### 2.5 Statistical analysis

The statistical data analysis was performed with IBM SPSS Statistics Version 20. First, one-way ANOVAs were used for exploring group differences in the meta references and graph indices (two-tailed, *p* < 0.05). The ANOVA had a factor Group (MSA-P, PD, and HC). *Post hoc* tests were used after observing significant group differences.

Second, partial correlation analyses were used for exploring the relationship between levodopa equivalent daily dose and the meta references and graph indices that exhibited group differences in MSA-P and PD groups, respectively (two-tailed, *p* < 0.05). The MDS-UPDRS Part III score was controlled. There was no covariate.

## 3 Results

### 3.1 Group differences in meta references

**Figure 2:**
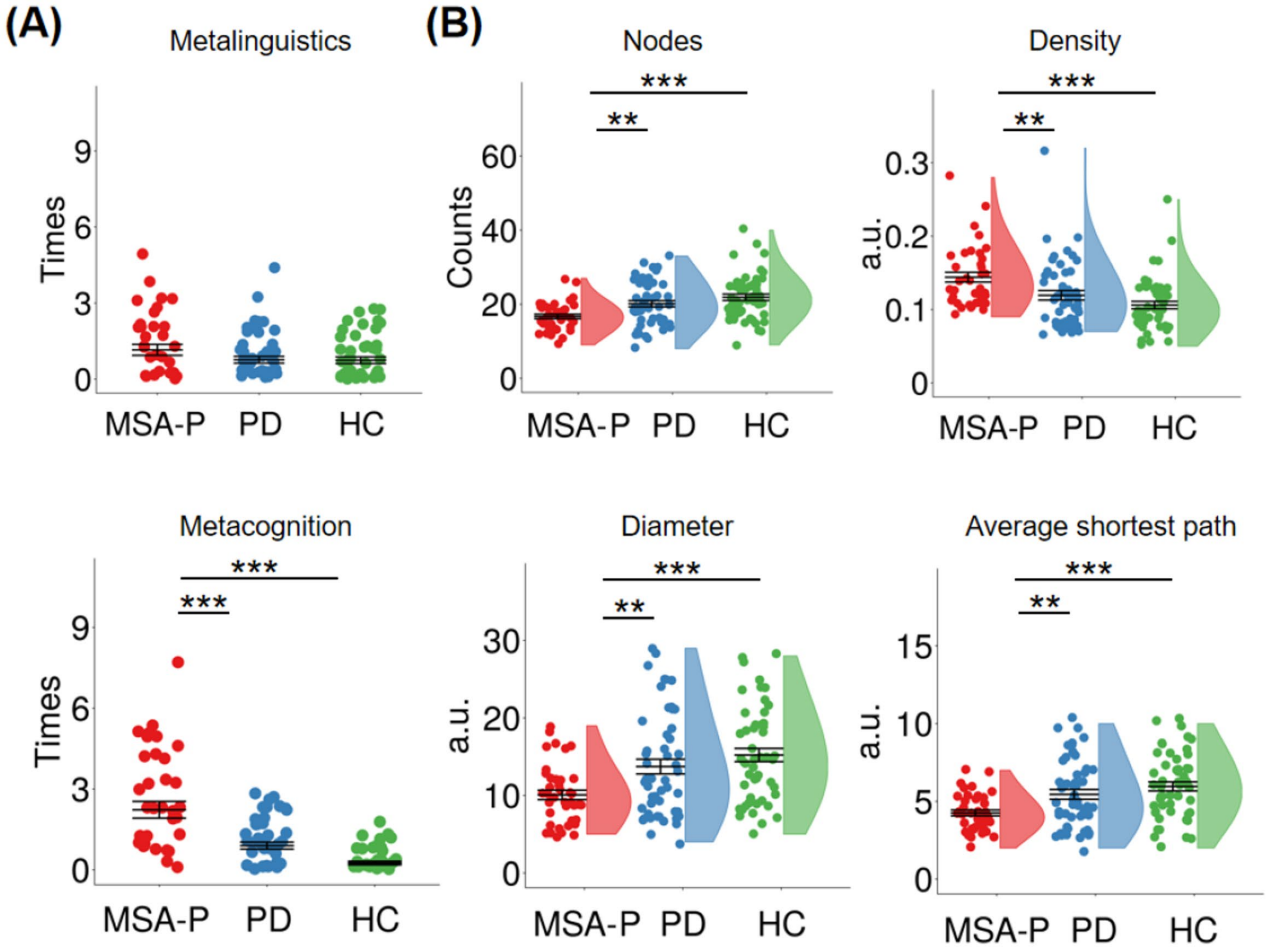
**(A)** Means and standard errors of metalinguistic and metacognitive references in parkinsonian-type multiple system atrophy patients (MSA-P), idiopathic Parkinson’s disease patients (PD), and healthy controls (HC). **(B)** Means and standard errors of nodes, density, diameter, and average shortest path in three groups. Asterisks (*) mean significant group differences between MSA-P and PD or HC in meta references and graph indices.

Figure 2A shows meta references in each group. Group difference was obtained in the metacognitive reference [*F*(2, 138) = 30.70, *p <* 0.001, η*_p_*^2^ = 0.31], but not in the metalinguistic reference [*F*(2, 138) = 1.97, *p =* 0.144, η*_p_*^2^ = 0.03]. Compared to PD patients and HC, the MSA-P patients mentioned more about their memory and remaining time (PD: *p <* 0.001; HC: *p <* 0.001).

### 3.2 Group differences in graph indices

Figure 2B shows graph indices in each group. Group differences were obtained in the nodes [*F*(2, 138) = 9.77, *p <* 0.001, η*_p_*^2^ = 0.12], density [*F*(2, 138) = 9.10, *p <* 0.001, η*_p_*^2^ = 0.12], diameter [*F*(2, 138) = 8.82, *p <* 0.001, η*_p_*^2^ = 0.11], and average shortest path [*F*(2, 138) = 10.08, *p <* 0.001, η*_p_*^2^ = 0.13]. Compared to PD patients and HC, the MSA-P group showed fewer nodes (PD: *p* = 0.004; HC: *p <* 0.001), higher density (PD: *p* = 0.007; HC: *p <* 0.001), smaller diameter (PD: *p* = 0.004; HC: *p <* 0.001), and smaller average shortest path (PD: *p* = 0.002; HC: *p <* 0.001). Specifically, MSA-P patients’ speech graphs were denser and smaller than PD patients and HC.

### 3.3 Effect of levodopa in MSA-P and PD patients

**Figure 3:**
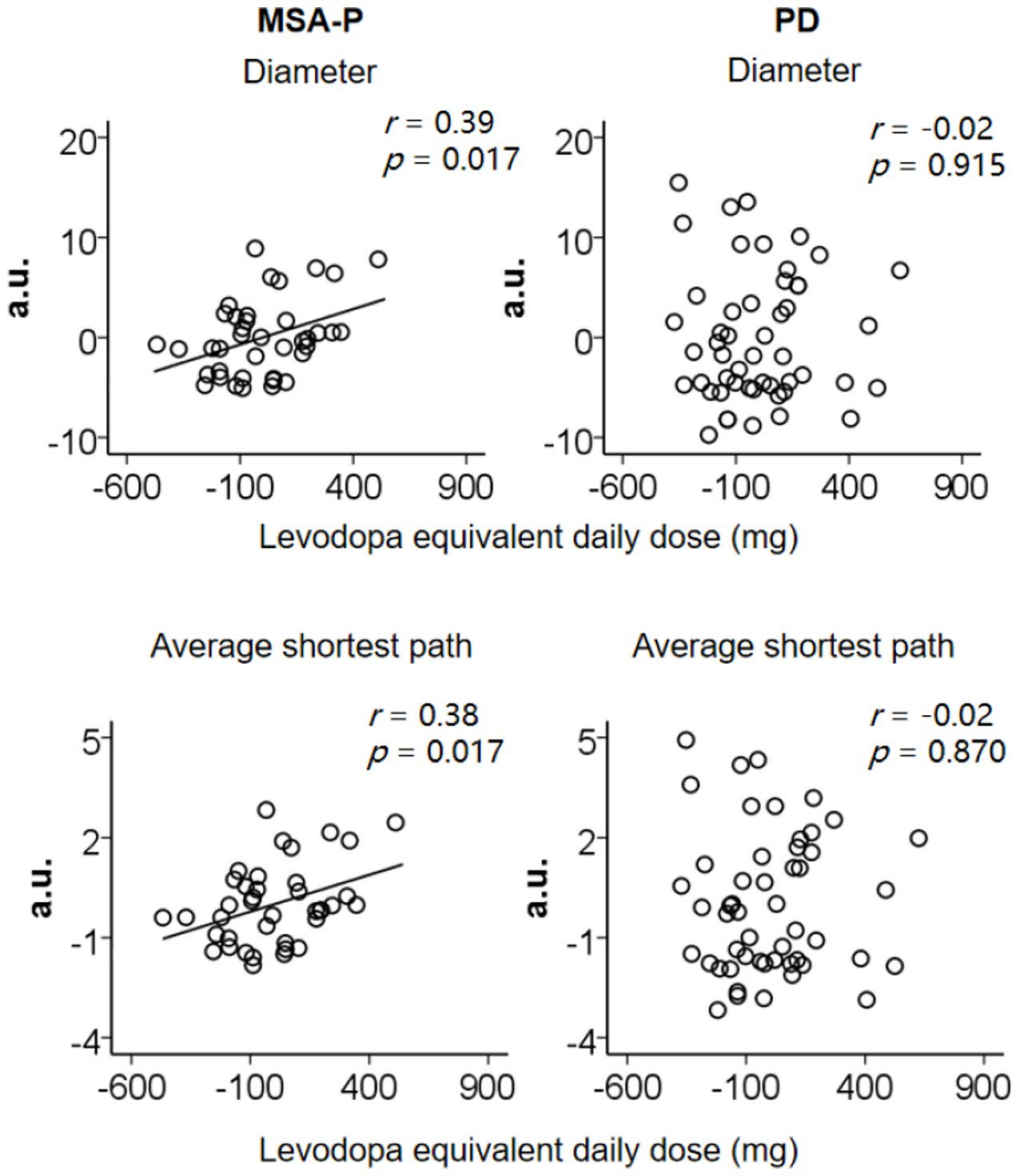
In MSA-P, the positive correlations between levodopa equivalent daily dose and diameter and average shortest path when the MDS-UPDRS part III score was controlled. In PD, there was no significant correlation. Values were demeaned.

Figure 3 shows the effect of levodopa on graph indices in MSA-P and PD patients, respectively. For the MSA-P patients, when the MDS-UPDRS Part III score was controlled, the positive correlations between levodopa equivalent daily dose and the diameter (*r* = 0.39, *p* = 0.017) and average shortest path (*r* = 0.38, *p* = 0.017) were obtained. It suggested that higher levodopa doses were associated with producing sparser and larger speech graphs in MSA-P patients. For the PD patients, we did not acquire the significant correlation between levodopa equivalent daily dose and diameter or average shortest path (*p*s>0.870).

There was no significant correlation between levodopa equivalent daily dose and other indices (*p*s>0.291).

## 4 Discussion

This study applied the graph theoretical analysis for investigating the topology of speech graphs in non-demented patients with MSA-P. Compared to PD patients and healthy adults, MSA-P patients produced denser and smaller speech graphs. Specifically, MSA-P patients’ speech graphs exhibited fewer nodes, higher density, smaller diameter, and smaller average shortest path in comparison to both PD patients and healthy adults. This study confirmed that graph theoretical analyses could differentiate MSA-P and PD, which is similar to a previous study. Šubert et al. (17) used the automated linguistic analysis of natural spontaneous speech and also achieved this goal. Importantly, in MSA-P patients, the positive correlations between levodopa equivalent daily dose and diameter and average shortest path were obtained. Particularly, there was no MSA-P patient who did not receive levodopa (levodopa equivalent daily dose = 0 mg/day). It suggested that higher levodopa doses were associated with producing sparser and larger speech graphs in MSA-P patients.

Additionally, the increased meta-references may be because of deficits in both lexical retrieval and executive control. Prefrontal cortex has long been considered essential for option generation (nodes) and executive functions. A fMRI study showed that the prefrontal cortex activation was related to option production in simple real-world scenarios (18). Enhanced left prefrontal activation has been observed in the word generation within a semantically similar category (19). Moreover, the left inferior frontal gyrus modulate the verbal responses production that satisfy the instruction and inhibit irrelevant words (20). It has been confirmed that executive control is realized by the prefrontal cortex that sends command signals to supervise and control the activities in other cortical and subcortical regions (21).

In addition, the basal ganglia also participated in speech initiation and programming. An electrophysiological research used microelectrode recordings and found that sensori-motor subthalamic neuronal activity was associated with generating speech programming during spoken sentence and syllable-repetition tasks (22). A MRI study investigated the contribution of basal ganglia on verbal fluency and exhibited that the caudate nucleus associated with language generation and the putamen activation improved the switching condition (23).

The speech topological abnormalities in MSA-P patients may result from aberrant connectivity of the frontal-basal ganglionic circuits compared to PD patients (24). A PET study confirmed that the frontal, temporal, parietal, and limbic hypometabolism was associated with cognitive decline in MSA patients (25). Another thin section MR study pointed out that the putaminal signal abnormalities and hyperintensities on proton density were more common in MSA-P patients compared to PD patients (26).

Neurochemical basis of speech graph is still unclear. A model suggested that the dopamine modulating fronto-striatal circuitry might relevant to the persistence and flexibility, respectively (27). To be specific, appropriate dopamine levels in prefrontal cortex promote persistent processes (words generation within a specific category or subcategory) along the mesocortical pathway, while appropriate dopamine levels in striatum accelerate flexible processes (words generation within different categories or subcategories) along the nigrostriatal pathway. Dual-state theory indicated that the prefrontal cortex with concentrated dopamine D1 receptor supports maintenance of item, while the striatum with concentrated dopamine D2 receptor favors flexibility of item (28). A previous study proposed that the levodopa, rather than dopamine agonist pramipexole, could enhance verbal fluency in PD patients (29). There also have conflicting results. For example, Subert et al. (30) found that PD patients who were treated with dopamine agonists showed improvement in sentence length, while who were treated with levodopa had no language amelioration. This study showed that levodopa could improve speech graphs in MSA-P patients. These results may be possibly by activating dopamine D1 receptors (31).

This study has limitations. First, in view of the small sample size of MSA patients with other clinical phenotypes and increasing global homogeneity, we only include patients with MSA-P, thus it’s impossible for this study to differentiate between MSA-P and other clinical phenotypes by graph theory-based analysis of semantic fluency data. Second, although we found the relationship between levodopa equivalent daily dose and graph indices, it cannot be regarded as a causal conclusion. Third, there were very few MSA-P patients took dopamine D2 receptor agonists, thus this study cannot examine the effect of activating dopamine D2 receptor on speech graphs. Future pharmacological researches should explore how antiparkinsonian medications affect speech topology in MSA patients.

## 5 Conclusion

This study applied graph theory to demonstrate the topological properties of speech graphs in MSA-P patients, indicating that MSA-P patients’ speech graphs were denser and smaller in comparison to PD patients and healthy adults. Moreover, higher levodopa doses were associated with producing sparser and larger speech graphs in MSA-P patients, indicating that the impaired speech topology can be partially restored by levodopa. Future studies should explore long-term successful treatments to achieve the improvement of speech topology in MSA-P patients.

## Data Availability

The original contributions presented in the study are included in the article/supplementary material, further inquiries can be directed to the corresponding author.
